# Hierarchical controller synthesis using ($\upgamma ,\updelta$)-Similarity

**DOI:** 10.1007/s00498-026-00437-z

**Published:** 2026-02-18

**Authors:** Armin Pirastehzad, Arjan van der Schaft, Bart Besselink

**Affiliations:** Bernoulli Institute for Mathematics, Computer Science and Artificial Intelligence, University of Groningen, 9700 AK Groningen, The Netherlands

**Keywords:** System abstraction, Hierarchical control synthesis, Approximate simulation, Compositional reasoning, 93A13, 93B50, 93B51, 93C05, 93D05, 93D25

## Abstract

We compensate for the scalability issues in controller synthesis by developing a hierarchical control scheme within the framework of ($\upgamma ,\updelta$)-similarity, which measures to what extent a potentially non-deterministic system satisfies specifications expressed as solution trajectories of a dynamical ‘specification’ system. This scheme synthesizes a controller for a non-deterministic ‘concrete’ system in three *hierarchical* steps. First, an ‘abstract’ system, which is a low-dimensional model of the concrete system, is obtained. Then, a controller is designed for the abstract system. At last, the abstract controller is refined into the concrete controller through an ‘interface’. To enable this, we introduce and characterize the notion of ($\upgamma ,\updelta$)-abstraction that utilizes an $\mathcal {L}_2$ approximation metric to measure the behavioral similarity of the concrete system and its abstraction in the presence of the interface. We utilize this characterization to propose a step-by-step procedure to construct the interface. We then synthesize the abstract controller and refine it into a concrete one.

## Introduction

Control of modern engineering systems has become exceedingly arduous as these systems have grown increasingly large and complex. Controller synthesis for such large-scale intricate systems suffers from scalability issues in the sense that existing design methods do not generally scale well with growing system dimension. In particular, many of these methods lead to controllers that have the same dimension as the system, which gives rise to expensive computations in synthesis and prohibitive costs in implementation. Furthermore, most existing design methods fail to address sophisticated specifications that the increasing requirements on the complex behavior of modern engineering systems require.

Lack of scalability in synthesis prompts the mobilization of *hierarchical* schemes [1] that decompose a complex synthesis problem that involves expensive computations into a number of simple ‘sub-problems’ of lower computational costs. Specifically, such schemes split the control of a high-dimensional *concrete* system into (1) *abstraction* through which a low-dimensional abstract model of the concrete system is obtained, (2) *design* where a controller is constructed for the abstract system, and (3) *refinement* by which the abstract controller is refined into the concrete controller using a so-called interface. Complexity of relevant control specifications, on the other hand, motivates the utilization of design techniques that express control specifications in terms of solution trajectories of a dynamical ‘specification’ system and accordingly address control synthesis in the context of *system comparison* [2]. In this paper, we develop a hierarchical control scheme that conducts synthesis in the context of ($\gamma ,\delta$)-similarity [3], which measures to what extent a system behaves similarly to a specification system.

Hierarchical schemes can be identified according to the abstraction method they utilize. Symbolic methods [4, 5] exploit system relations, such as (bi)simulation [6], approximate (bi)simulation [7], and approximate alternating (bi)simulation [2], to obtain a finite, discrete-state abstraction of the concrete system. Such methods, however, suffer from the curse of dimensionality in the sense that high-dimensional systems admit abstractions with intractably large number of states. This motivated the development of methods that make use of (robust) simulation functions [8–15] to construct continuous abstractions. These methods measure the behavioral similarity of a concrete system and its abstraction in an approximation metric that utilizes the $\mathcal {L}_\infty$ signal norm. Such approximation metric, however, is incompatible with many analytic and synthetic tools in control theory as they often employ the $\mathcal {L}_2$ signal norm, e.g., in robust control [16] or dissipativity theory [17, 18].

Design techniques that express specifications in terms of input-output trajectories of a dynamical specification system exploit system relations, such as (bi)simulation [6] and approximate (bi)simulation [19], to address control synthesis in the context of system comparison [20, 21]. Concerned with exact input-output equivalence of systems, control synthesis on the basis of (bi)simulation fails to address the case where the external behavior of a system and its corresponding specification are *close* but not identical. Control synthesis on the basis of approximate (bi)simulation resolves these limitations as it relaxes the requirement on exact equivalence. In particular, such methods compare the external behavior of a system and its specification in an approximation metric that utilizes the $\mathcal {L}_\infty$ signal norm, which hinders their use in control theory as stated above.

The goal of this paper is to develop a hierarchical control framework that conducts synthesis in the context of ($\gamma ,\delta$)-similarity, which utilizes the $\mathcal {L}_2$ signal norm to measure to what extent the external behavior of a system is similar to that of a specification system. The main contributions of this paper are listed as follows.

First, by extending the notion of ($\gamma ,\delta$)-similarity to control systems, we conceive a notion of abstraction that enables us to identify the abstract system. We introduce the notion of ($\gamma ,\delta$)-abstraction, which measures to what extent the *controlled* concrete system behaves similarly to a linear abstract system in the presence of an interface, which is a dynamical system that will be used later for controller refinement. In this setting, the concrete system is *non-deterministic* as its solution trajectories may depend on unknown disturbances. While the notion of ($\gamma ,\delta$)-abstraction measures the behavioral similarity of control systems in an approximate sense, it is different from the notion of alternating approximate simulation as it makes use of the $\mathcal {L}_2$ signal norm to measure similarity. Specifically, by formulating behavioral similarity in terms of the sensitivity of the output difference to the input signals, the notion of ($\gamma ,\delta$)-abstraction utilizes the $\mathcal {L}_2$ signal norm to characterize a bound on the output difference in terms of the input signals. This, as a result, makes ($\gamma ,\delta$)-abstraction compatible with many effective analytic and synthetic tools developed in control theory, e.g., $H_\infty$ synthesis [17, 22].

Second, we draw inspiration from $H_\infty$ synthesis (e.g., [22]) to characterize ($\gamma ,\delta$)-abstraction as a linear matrix inequality (LMI) feasibility problem that is completely in terms of the system matrices. We then propose a step-by-step procedure through which we utilize the solution of the LMI feasibility problem to construct the interface.

Third, we formalize the problem of abstract controller synthesis where we obtain the abstract controller as a solution of an LMI feasibility problem. We then use the interface to refine this controller into the concrete one. In particular, the interface takes information from the concrete system, the abstract system, and the abstract controller to generate the concrete control signal. After refinement, we conduct a thorough analysis to study the stability properties of the controlled concrete system (that contains the concrete system and the concrete controller). We accordingly propose a step-by-step algorithm for hierarchical control of the concrete system.

The rest of this paper is organized as follows. In Sect. 2, we address specification verification in the context of ($\gamma ,\delta$)-similarity. Then, in Sect. 3, we rigorously state the problem of hierarchical control synthesis using ($\gamma ,\delta$)-similarity. We introduce and characterize the notion of ($\gamma ,\delta$)-abstraction in Sect. 4, where we also propose a procedure to construct the interface. In Sect. 5, we formalize the abstract synthesis problem and characterize the abstract controller. Subsequently, in Sect. 6, after conducting stability analysis, we propose an algorithm for hierarchical control synthesis. We then demonstrate our results in an illustrative example in Sect. 7 and, finally, conclude the paper in Sect. 8.

***Notation***

Given a matrix *M*, we denote by $M^\perp$ any matrix whose columns form a basis for $\operatorname {Ker} M$, *i.e.,* $MM^\perp = 0$. Also, for any square matrix *M*, we define the operator $\langle \cdot \rangle _s$ as $\langle M \rangle _s = M+M^\top$. Given $x\in \mathbb {R}^n$, we define the Euclidean norm as $|x| = (x^\top x)^{1/2}$. Then, for a positive (semi-)definite matrix $M \in \mathbb {R}^{n\times n}$, the weighted Euclidean (semi-)norm is defined as $|x|_M = (x^\top M x)^{1/2}$. Accordingly, we define the function space $\mathcal {L}_2 = \{u: [0,\infty ) \rightarrow \mathbb {R}^n \ | \ \int _{0}^{\infty }|u(t)|^2\textrm{d}t< \infty \}$, endowed with the norm $\left\Vert u\right\Vert = (\int _{0}^{\infty }|u(t)|^2\textrm{d}t)^{1/2}$. Lastly, for a positive (semi-)definite matrix $M \in \mathbb {R}^{n\times n}$, we define the weighted norm as $\left\Vert u\right\Vert _M = (\int _{0}^{\infty }|u(t)|_M^2\textrm{d}t)^{1/2}$.

## Specification verification by ($\upgamma ,\updelta$)-similarity

Consider the continuous-time linear system

1 $$\begin{aligned} \boldsymbol{\Sigma }: \left\{ \begin{aligned} \dot{x}&= Ax + Ew+Fd,\\ z&= Hx, \end{aligned} \right. \end{aligned}$$

with state $x\in \mathbb {R}^{n}$, external input $w\in \mathbb {R}^{k}$, disturbance $d\in \mathbb {R}^{q}$, and output $z\in \mathbb {R}^{p}$. We assume that (1) is 0-asymptotically stable, *i.e.,* the system is asymptotically stable in the absence of external inputs and disturbances. Equivalently, the state matrix *A* is Hurwitz. By *z*(*t*; *w*, *d*), we denote the output solution, at time *t*, of (1) for initial condition $x(0) = 0$, external input *w*, and disturbance *d*. We note that (1) is non-deterministic in the sense that its solution trajectories depend not only on the external input *w*, but also on the unknown disturbance *d*.

We define specifications on the input-output behavior of (1) in terms of the input-output trajectories of the *specification* system

2 $$\begin{aligned} \boldsymbol{S}: \left\{ \begin{aligned} \dot{x}_s&= A_sx_s+E_sw_s+F_sd_s,\\ z_s&= H_sx_s, \end{aligned} \right. \end{aligned}$$

where $x_s\in \mathbb {R}^{n_s}$, $w_s\in \mathbb {R}^{k}$, $d_s\in \mathbb {R}^{q_s}$, and $z_s\in \mathbb {R}^{p}$ respectively represent system state, external input, driving variable, and output. We also assume that (2) is 0-asymptotically stable, *i.e.,* the state matrix $A_s$ is Hurwitz. We use notation similar to that of (1) to denote the output solution to (2).

In (1), the input *w* and the output *z* are regarded as external variables that allow for system interconnection, whereas the disturbance *d* accounts for non-determinism. Correspondingly, in (2), the input $w_s$ and output $z_s$ are external variables that enable interconnection among specifications. In view of the fact that (2) captures specifications, $d_s$ is a driving variable responsible for generating the class of ‘desirable’ input-output trajectories that form the specification.

We measure to what extent the system (1) satisfies the specification (2) by comparing the behavioral *similarity* of (1) and (2) according to the notion of ($\gamma ,\delta$)-similarity, which is taken from [3].

### Definition 1

For $\gamma ,\delta >0$, the specification $\boldsymbol{S}$ is said to be ($\gamma ,\delta$)-similar to the system $\boldsymbol{\Sigma }$, denoted by $\boldsymbol{\Sigma } \preccurlyeq _{\gamma ,\delta } \boldsymbol{S}$, if there exist constants $\varepsilon ,\eta ,\mu >0$ such that for every external input $w,w_s\in \mathcal {L}_2$ and every disturbance $d\in \mathcal {L}_2$, there exists a driving variable $d_s\in \mathcal {L}_2$ such that

3 $$\begin{aligned} \begin{aligned} \left\Vert z-z_s\right\Vert ^2\le \gamma \left\Vert w-w_s\right\Vert ^2+(\delta -\varepsilon )\left\Vert \begin{bmatrix} w\\ w_s \end{bmatrix}\right\Vert ^2+(\mu -\varepsilon ) \left\Vert d\right\Vert ^2 - \eta \left\Vert d_s\right\Vert ^2, \end{aligned} \end{aligned}$$

where $$z(t) = z(t;w,d)$$ and $$z_s(t) = z_s(t;w_s,d_s)$$.

The notion of ($\gamma ,\delta$)-similarity in Definition 1 measures the similarity of the trajectories of $\boldsymbol{\Sigma }$ and $\boldsymbol{S}$ in terms of their input-output behavior. In (3), the (small) parameter $\varepsilon$ is included only for technical reasons, which will become evident when we characterize $(\gamma ,\delta )$-similarity. The parameter $\gamma$, however, measures to what extent a deviation in external inputs leads to a deviation in outputs, whereas the parameter $\delta$ accounts for how each external input affects this deviation, which is essential as it also provides a bound on output deviation in the case where $w = w^s$. The parameter $\mu$, on the other hand, captures the effect of the disturbance *d* in the output deviation, while the parameter $\eta$ specifies how the driving variable $d^s$ contributes to this deviation. More specifically, when input signals *w* and $w^s$ are given, Definition 1 states that for any trajectory of $\boldsymbol{\Sigma }$ (now determined by *d*), there exists a trajectory of $\boldsymbol{S}$ (through the choice of $d^s$) that approximates it according to (3). Definition 1 therefore indicates the ability of $\boldsymbol{S}$ to *approximately* generate the input-output trajectories of $\boldsymbol{\Sigma }$, *i.e.,* ($\gamma ,\delta$)-similarity can be interpreted as a notion that measures to what extent the input-output behavior of $\boldsymbol{\Sigma }$ is contained that of $\boldsymbol{S}$.

### Remark 1

Despite measuring the behavioral similarity of a system (1) and its specification (2), the notion of ($\gamma ,\delta$)-similarity does not require the external inputs to the two systems to be the same, *i.e.,* $w_s$ and *w* are not asked to be the same. This allows for the extension of ($\gamma ,\delta$)-similarity from single components/specifications to their interconnections. Namely, in such cases the external inputs *w* and $w_s$ of a given component/specification might result from the output of another component/specification and are not necessarily the same. This flexibility in the notion of ($\gamma ,\delta$)-similarity allows for the characterization of ($\gamma ,\delta$)-similarity for interconnected systems/specifications in terms of ($\gamma ,\delta$)-similarity for their constituting components (see [3, Section V] for further details).$\triangle$

Measuring to what extent the input-output behavior of $\boldsymbol{\Sigma }$ is contained in that of $\boldsymbol{S}$, the notion of ($\gamma ,\delta$)-similarity measures how *accurately* $\boldsymbol{\Sigma }$ satisfies the specifications given by $\boldsymbol{S}$, see [23] for details.

The notion of ($\gamma ,\delta$)-similarity can be characterized as a matrix inequality that is completely in terms of the parameters of $\boldsymbol{\Sigma }$ and $\boldsymbol{S}$. Through a few simple algebraic manipulations, (3) can be easily recast as

4 $$\begin{aligned} \left\| \begin{bmatrix} z - z_s\\ d_s \end{bmatrix}\right\| _{R(\eta )}^ 2 \le \left\| \begin{bmatrix} w\\ w_s\\ d \end{bmatrix}\right\| _{Q(\gamma ,\delta ,\mu )}^2 - \varepsilon \left\| \begin{bmatrix} w\\ w_s\\ d \end{bmatrix}\right\| ^ 2, \end{aligned}$$

where

5 $$\begin{aligned} Q(\gamma ,\delta ,\mu ) = \begin{bmatrix} (\gamma +\delta )I& -\gamma I& 0\\ -\gamma I& (\gamma +\delta )I& 0\\ 0& 0& \mu I \end{bmatrix}, \quad R(\eta ) = \begin{bmatrix} I& 0\\ 0& \eta I \end{bmatrix}. \end{aligned}$$

It thus follows from Definition 1 that the notion of ($\upgamma ,\updelta$)-similarity seeks a driving variable $d_s\in \mathcal {L}_2$ that establishes (4) for all external inputs $w,w_s\in \mathcal {L}_2$ and disturbances $d\in \mathcal {L}_2$. Such representation enables the formulation of ($\upgamma ,\updelta$)-similarity in terms of *strict* dissipativity [3, Lemma 4], where the (small) parameter $\varepsilon$ in (3) guarantees this *strictness*. Then, by defining the *extended* matrices

$$\begin{aligned} \begin{aligned} A_e&= \begin{bmatrix} A& 0\\ 0& A_s \end{bmatrix},\!\!&B_e&=\begin{bmatrix} 0\\ F_s \end{bmatrix},&E_e&=\begin{bmatrix} E& 0& F\\ 0& E_s& 0 \end{bmatrix},&H_e&= \begin{bmatrix} H& -H_s\\ 0& 0 \end{bmatrix},&D_e&=\begin{bmatrix} 0\\ I \end{bmatrix}, \end{aligned} \end{aligned}$$

this dissipativity-based formulation yields the following characterization, see [3, Theorem 2] for details.

### Proposition 1

For $\gamma ,\delta >0$, $\boldsymbol{\Sigma }\preccurlyeq _{\gamma ,\delta }\boldsymbol{S}$ if and only if there exist matrices $X\succ 0$, $F_e$, and scalars $\hat{\mu },\hat{\eta }>0$ such that

6 $$\begin{aligned} \begin{bmatrix} \langle X(A_e+B_eF_e)\rangle _s& XE_e& (H_e+D_eF_e)^\top \\ E_e^\top X& -Q(\gamma ,\delta ,\hat{\mu })& 0\\ H_e+D_eF_e& 0& -R(\hat{\eta }) \end{bmatrix}\prec 0, \end{aligned}$$

where matrices *Q* and *R* are defined as in (5).

### Remark 2

Despite the fact that Definition 1 does not make any prior assumptions on the structure of $d_s$, it can be concluded from Proposition 1 that one may always find the signal $d_s$ as a static state feedback, *i.e.,* $d_s = F_ex_e$. This allows one to restrict the search for $d_s$ to signals that can be expressed in terms of static state feedback. $\triangle$

## The hierarchical synthesis problem

Concerned with the situation where a system fails to satisfy a specification with a desirable accuracy, we are interested in the problem of synthesizing a controller that enforces a system to guarantee a specification in the context of ($\gamma ,\delta$)-similarity. More specifically, given a *concrete* system and a specification system (that captures the input-output specifications), we aim to design a controller such that the specification system is ($\gamma ,\delta$)-similar to the closed-loop dynamics that contains the concrete system and the controller.

We therefore consider the *concrete* system

7 $$\begin{aligned} \boldsymbol{\Sigma }_c: \left\{ \begin{aligned} \dot{x}_c&= A_cx_c+ B_cu_c + E_cw_c+F_cd_c,\\ z_c&= H_cx_c, \end{aligned} \right. \end{aligned}$$

with state $x_c\in \mathbb {R}^{n_c}$, control input $u_c\in \mathbb {R}^{m_c}$, external input $w_c\in \mathbb {R}^{k_c}$, disturbance $d_c\in \mathbb {R}^{q_c}$, and controlled output $z_c\in \mathbb {R}^{p_c}$. By $z_c(t; u_c,w_c,d_c)$, we denote the output solution, at time *t*, of (7) for initial condition $x_c(0) = 0$, control input $u_c$, external input $w_c$, and disturbance $d_c$. We crucially assume that output $z_c$ and external input $w_c$ can be *measured*.

While the system state $x_c$ is an internal variable, the external input $w_c$ and the controlled output $z_c$ are external variables through which (7) interacts with its environment, *i.e.,* they are external variables that allow system interconnection. The control input $u_c$, on the other hand, is to be designed to control the behavior of $z_c$. The disturbance $d_c$ accounts for non-determinism in the sense that trajectories do not solely depend on the control input $u_c$ and the external input $w_c$.

**Fig. 1:**
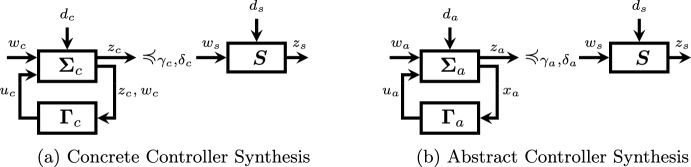
Controller synthesis for the concrete and the abstract systems

Given a specification $\boldsymbol{S}$ and constants $\gamma _c,\delta _c>0$, our objective is to synthesize a dynamic (measurement) output feedback controller $\boldsymbol{\Gamma }_c$ (see Fig. 1a) for a complex, high-dimensional concrete system $\boldsymbol{\Sigma }_c$ such that

I. The controlled concrete system, *i.e.,* the closed-loop system that is composed of the concrete system $\boldsymbol{\Sigma }_c$ and the concrete controller $\boldsymbol{\Gamma }_c$, is 0-asymptotically stable;
II. The specification $\boldsymbol{S}$ is ($\gamma _c,\delta _c$)-similar to the controlled concrete system.

Synthesis of $\boldsymbol{\Gamma }_c$, however, suffers from scalability issues as design methods generally do not scale well with system dimension. In particular, the existing method for the synthesis of $\boldsymbol{\Gamma }_c$ leads to a controller of the same order as the system [23], which requires expensive computations in synthesis and imposes prohibitive costs in implementation. To compensate for the scalability issues, we synthesize the concrete controller $\boldsymbol{\Gamma }_c$ according to an *hierarchical* control scheme that splits the synthesis procedure into the following consecutive steps.

*Abstraction:* We first construct an *abstract* system, which is a low-dimensional, coarser model of the concrete system (7) that admits a *richer* input-output behavior, *i.e.,* the abstract system (approximately) contains the input-output trajectories of the concrete system. We construct the abstract system as a system of the form

8 $$\begin{aligned} \boldsymbol{\Sigma }_a: \left\{ \begin{aligned} \dot{x}_a&= A_ax_a + B_au_a + E_aw_a + F_ad_a,\\ z_a&= H_ax_a, \end{aligned} \right. \end{aligned}$$

with state $x_a \in \mathbb {R}^{n_a}$, control input $u_a \in \mathbb {R}^{m_a}$, external input $w_a \in \mathbb {R}^{k_a}$, driving signal $d_a \in \mathbb {R}^{q_a}$, and controlled output $z_a\in \mathbb {R}^{p_a}$. We use notation similar to that of (7) to denote the output solution to (8). While we respectively regard $w_a$ and $u_a$ as ‘abstract’ counterparts to $w_c$ and $u_c$ (*i.e.,* $w_a$ and $z_a$ are external variables that allow system interconnection), we do not treat $d_a$ as a physical disturbance, but rather as a driving variable that allows the abstract system (8) to have richer input-output behavior than the concrete system (7).

*Design:* We then design an abstract controller $\boldsymbol{\Gamma }_a$ (see Fig. 1b such that 1) the controlled abstract system that contains $\boldsymbol{\Sigma }_a$ and $\boldsymbol{\Gamma }_a$ is 0-asymptotically stable, and 2) for some suitable $\gamma _a,\delta _a>0$, the specification system $\boldsymbol{S}$ is ($\gamma _a,\delta _a$)-similar to the controlled abstract system. Considering that the abstract system (8) is a low-dimensional *virtual* system whose states are *always* available, such controller can be designed on the basis of state feedback, which is computationally efficient.

*Refinement:* We finally refine the synthesized abstract controller $\boldsymbol{\Gamma }_a$ into a concrete controller $\boldsymbol{\Gamma }_c$ for the concrete system. Using an *interface*, we refine $\boldsymbol{\Gamma }_a$ into $\boldsymbol{\Gamma }_c$ in such a way that the resulting controlled concrete system (that contains $\boldsymbol{\Sigma }_c$ and $\boldsymbol{\Gamma }_c$) satisfies conditions (I) and (II). We construct the interface as a dynamic system of the form

9 $$\begin{aligned} \boldsymbol{I}:\left\{ \begin{aligned} \dot{x}_i&= A_ix_i + B_iu_i,\\ y_i&= C_ix_i + D_iu_i, \end{aligned}\right. \end{aligned}$$

where $x_i\in \mathbb {R}^{n_i}$, $u_i = \operatorname {col}(z_c,x_a,u_a)$, and $y_i = \operatorname {col}(u_c,d_a)$. The interface therefore takes feedback from the concrete system and the controlled abstract system (which is the composition of $\boldsymbol{\Sigma }_a$ and $\boldsymbol{\Gamma }_a$) to generate the control input $u_c$ and the driving signal $d_a$. In particular, the interface (9) generates $u_c$ and $d_a$ on the basis of taking a feedback from the measurement output $z_c$, the abstract state $x_a$, and the abstract control input $u_a$.

**Fig. 2:**
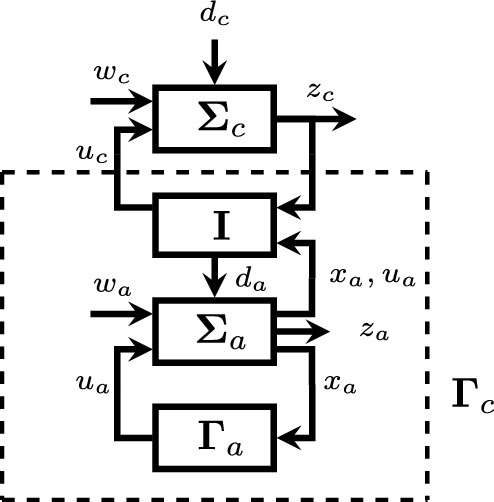
We hierarchically construct the concrete controller $\boldsymbol{\Gamma }_c$ as the composite system that contains the abstract system $\boldsymbol{\Sigma }_a$, the abstract controller $\boldsymbol{\Gamma }_a$, and the interface $\boldsymbol{I}$. We will later show that such concrete controller $\boldsymbol{\Gamma }_c$ simultaneously achieves conditions (I) and (II) for a particular choice of $w_a$ that depends on the external inputs $w_c$, which highlights the incorporation of $w_c$ as a part of the feedback input to $\boldsymbol{\Gamma }_c$

We therefore establish a hierarchical synthesis framework that constructs the concrete controller $\boldsymbol{\Gamma }_c$ according to the architecture in Fig. 2, where $\boldsymbol{\Gamma }_c$ is derived as the composite system that contains the abstract system $\boldsymbol{\Sigma }_a$, the abstract controller $\boldsymbol{\Gamma }_a$, and the interface $\boldsymbol{I}$.

## ($\upgamma ,\updelta$)-abstraction

We conceive a notion of system abstraction according to which we identify the abstract system. We then give a characterization of this notion that enables us to construct the interface.

To define a notion of abstraction, we collect the dynamics of the concrete system (7) and a given abstract system (8). We define $x_e:=\operatorname {col}(x_c,x_a)$, $u_e:=\operatorname {col} (u_c,d_a)$, $w_e:= \operatorname {col}(w_c,w_a,d_c,u_a)$, $y_e:=\operatorname {col}(z_c,x_a,u_a)$, and $z_e:=\operatorname {col}(z_c-z_a,d_a)$ to obtain the *extended* dynamics

10 $$\begin{aligned} \begin{aligned} \dot{x}_e&= A_ex_e+B_eu_e+E_ew_e,\\ y_e&= C_ex_e + D_ew_e,\\ z_e&= H_ex_e+K_eu_e, \end{aligned} \end{aligned}$$

where

11 $$\begin{aligned} A_e&= \begin{bmatrix} A_c& 0\\ 0& A_a \end{bmatrix},&B_e&=\begin{bmatrix} B_c& 0\\ 0& F_a \end{bmatrix},&C_e&=\begin{bmatrix} H_c& 0\\ 0& I\\ 0& 0 \end{bmatrix},&D_e&=\begin{bmatrix} 0& 0& 0& 0\\ 0& 0& 0& 0\\ 0& 0& 0& I \end{bmatrix}, \nonumber \\ E_e&=\begin{bmatrix} E_c& 0& F_c& 0\\ 0& E_a& 0& B_a \end{bmatrix},&H_e&=\begin{bmatrix} H_c& -H_a\\ 0& 0 \end{bmatrix},&K_e&=\begin{bmatrix} 0& 0\\ 0& I \end{bmatrix}. \end{aligned}$$

Note that the extended system (10) does not include any interaction between $\boldsymbol{\Sigma }_c$ and $\boldsymbol{\Sigma }_a$. We let this interaction be determined by the interface (9). In particular, choosing $u_e$ according to (9), *i.e.,* taking $u_e = y_i$, we let $x_{cl} = \operatorname {col}(x_e,x_i)$ and $z_{cl} = z_e$ to obtain the *closed-loop* dynamics

12 $$\begin{aligned} \begin{aligned} \dot{x}_{cl}&= A_{cl}x_{cl} + E_{cl}w_e,\\ z_{cl}&= H_{cl}x_{cl} + G_{cl}w_e, \end{aligned} \end{aligned}$$

where

13 $$\begin{aligned} \begin{aligned} A_{cl}&=\begin{bmatrix} A_e+B_eD_iC_e& B_eC_i\\ B_iC_e& A_i \end{bmatrix},&E_{cl}&= \begin{bmatrix} E_e+B_eD_iD_e\\ B_iD_e \end{bmatrix},\\ H_{cl}&=\begin{bmatrix} H_e+K_eD_iC_e&K_eC_i \end{bmatrix},&G_{cl}&= K_eD_iD_e. \end{aligned} \end{aligned}$$

The closed-loop dynamics (12) now describes the evolution of the solution trajectories of $\boldsymbol{\Sigma }_c$ and $\boldsymbol{\Sigma }_a$ when $u_c$ and $d_a$ are chosen according to (9), *i.e.,* when $u_c$ and $d_a$ are generated by the interface $\boldsymbol{I}$. We use the closed-loop dynamics (12) to conceive the notion of ($\gamma ,\delta$)-abstraction that identifies the abstract system $\boldsymbol{\Sigma }_a$.

### Definition 2

Given $\boldsymbol{\Sigma }_c$ and $\boldsymbol{\Sigma }_a$, for $\gamma ,\delta >0$, $\boldsymbol{\Sigma }_a$ is said to be a ($\gamma ,\delta$)-abstraction of $\boldsymbol{\Sigma }_c$ if there exist an interface (9) and constants $\varepsilon ,\mu ,\zeta ,\eta >0$ such that the dynamics (12) is 0-asymptotically stable and for every external input $w_c,w_a\in \mathcal {L}_2$, every control input $u_a\in \mathcal {L}_2$, and every disturbance $d_c\in \mathcal {L}_2$, we have that

14 $$\begin{aligned} \left\Vert z_c-z_a\right\Vert ^2 \le&\ \gamma \left\Vert w_c-w_a\right\Vert ^2+(\delta -\varepsilon )\left\Vert \begin{bmatrix} w_c\\ w_a \end{bmatrix}\right\Vert ^2 \\&+(\mu -\varepsilon )\left\Vert d_c\right\Vert ^2+(\zeta -\varepsilon )\left\Vert u_a\right\Vert ^2 - \eta \left\Vert d_a\right\Vert ^2.\nonumber \end{aligned}$$

Comparing the solution trajectories of $\boldsymbol{\Sigma }_c$ and $\boldsymbol{\Sigma }_a$ in the presence of $\boldsymbol{I}$, the notion of ($\gamma ,\delta$)-abstraction measures to what extent the *controlled* input-output behavior of the concrete system is *similar* to that of the abstract system. It follows from Definition 2 that the interface $\boldsymbol{I}$ generates the concrete control $u_c$ and the driving signal $d_a$ such that for any controlled trajectory of $\boldsymbol{\Sigma }_a$ (determined by $w_a$, $u_a$, and $d_a$ that is provided by $\boldsymbol{I}$), there exists a controlled trajectory of $\boldsymbol{\Sigma }_c$ (determined by $w_c$, $u_c$ that is provided by $\boldsymbol{I}$, and $d_c$) that approximates it according to (14). In (14), the inclusion of $\varepsilon$ is merely for technical reasons that will be explained later. The parameter $\gamma$ indicates to what extent a dissimilarity in external inputs affects the output deviation, whereas the parameter $\delta$ captures the effect that each external input has in the output deviation. The parameter $\mu$, on the other hand, measures the impact of non-determinism in the output deviation, while the parameter $\zeta$ manifests the contribution of the abstract control in the output deviation.

**Fig. 3:**
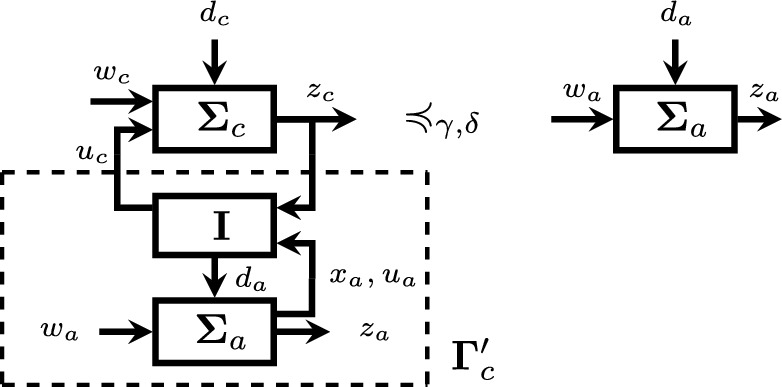
The uncontrolled abstract system is ($\gamma ,\delta$)-similar to the controlled concrete system

### Remark 3

Comparing the controlled input-output behavior of systems, the notion of ($\gamma ,\delta$)-abstraction generalizes ($\gamma ,\delta$)-similarity to control systems. In fact, ($\gamma ,\delta$)-abstraction implies ($\gamma ,\delta$)-similarity as a special case. In the absence of abstract control, *i.e.,* for $u_a = 0$, it immediately follows from Definition 2 that there exist $\varepsilon ,\mu ,\eta >0$ such that for every $w_c,w_a\in \mathcal {L}_2$ and every $d_c\in \mathcal {L}_2$, there exists a $d_a \in \mathcal {L}_2$ (that is obtained according to (9)) such that

$$\begin{aligned} \left\Vert z_c-z_a\right\Vert ^2 \le \gamma \left\Vert w_c-w_a\right\Vert ^2+(\delta -\varepsilon )\left\Vert \begin{bmatrix} w_c\\ w_a \end{bmatrix}\right\Vert ^2+(\mu -\varepsilon )\left\Vert d_c\right\Vert ^2 - \eta \left\Vert d_a\right\Vert ^2, \end{aligned}$$

which, by Definition 1, indicates that the interface $\boldsymbol{I}$ generates $u_c$ such that the *uncontrolled* abstract system is ($\gamma ,\delta$)-similar to the *controlled* concrete system. This is reminiscent to a synthesis problem in the sense that we may figuratively treat the uncontrolled $\boldsymbol{\Sigma }_a$ as a specification and consider the composite system that contains $\boldsymbol{I}$ and $\boldsymbol{\Sigma }_a$ as some controller $\boldsymbol{\Gamma }_c'$ for $\boldsymbol{\Sigma }_c$, see Fig. 3. This is in accordance with the internal model principle [24] as the controller contains the specification which generates the desirable input-output trajectories. $\triangle$

The notion of ($\gamma ,\delta$)-abstraction facilitates controller synthesis in the sense that it allows for the replacement of a high-dimensional system, which is computationally prohibitive to control, with a low-dimensional abstract system, which, by contrast, is computationally cheap to control.

### Characterization

We give an effective characterization of the notion of ($\gamma ,\delta$)-abstraction that enables us to construct the interface. For this purpose, we obtain an alternative formulation of (14) in terms of the closed-loop dynamics (12). Defining the matrices

15 $$\begin{aligned} Q(\mu ,\zeta ) = \begin{bmatrix} (\gamma +\delta )I& -\gamma I& 0& 0\\ -\gamma I&  (\gamma +\delta )I& 0& 0\\ 0& 0& \mu I& 0\\ 0& 0& 0& \zeta I \end{bmatrix}, \quad R(\eta ) =\begin{bmatrix} I& 0\\ 0& \eta I \end{bmatrix}, \end{aligned}$$

we rewrite (14) as

16 $$\begin{aligned} {{\int }}_{0}^\infty \begin{bmatrix} w_e(\tau )\\ z_{cl}(\tau ) \end{bmatrix}^\top \begin{bmatrix} -Q(\mu ,\zeta )& 0\\ 0& R(\eta ) \end{bmatrix}\begin{bmatrix} w_e(\tau )\\ z_{cl}(\tau ) \end{bmatrix}\textrm{d}\tau \le -\varepsilon \left\Vert w_e\right\Vert ^2. \end{aligned}$$

Based on this formulation, we obtain the following characterization of ($\gamma ,\delta$)-similarity.

#### Proposition 2

For $\gamma ,\delta >0$, $\boldsymbol{\Sigma }_a$ is a ($\gamma ,\delta$)-abstraction of $\boldsymbol{\Sigma }_c$ if and only if there exist matrices $X \succ 0$, $A_i$, $B_i$, $C_i$, $D_i$ and constants $\hat{\mu },\hat{\zeta },\hat{\eta }>0$ such that

17 $$\begin{aligned} \begin{bmatrix} A_{cl}^\top X+XA_{cl} & XE_{cl}& H_{cl}^\top \\ E_{cl}^\top X& -Q(\hat{\mu },\hat{\zeta })& G_{cl}^\top \\ H_{cl}& G_{cl}& -R(\hat{\eta }) \end{bmatrix} \prec 0, \end{aligned}$$

where *Q* and *R* are now given as in (15). In this case, (14) is satisfied with $\mu = \hat{\mu }$, $\zeta = \hat{\zeta }$, and $\eta = \hat{\eta }^{-1}$.

#### Proof

To show necessity, suppose $\boldsymbol{\Sigma }_a$ is a ($\gamma ,\delta$)-abstraction of $\boldsymbol{\Sigma }_c$. This, by Definition 2, indicates that there exists matrices $A_i$, $E_i$, $C_i$, $D_i$, and constants $\varepsilon ,\mu ,\zeta ,\eta >0$ such that (12) is 0-asymptotically stable and for every $w_c,w_a\in \mathcal {L}_2$, $u_a\in \mathcal {L}_2$, and $d_c\in \mathcal {L}_2$, (14) holds. After writing (14) as (16), we conclude from [17, Proposition 3.9] that there exists a matrix $X\succ 0$ such that for $\hat{\mu }=\mu$, $\hat{\zeta }=\zeta$, and $\hat{\eta }=\eta ^{-1}$, (17) holds.

To show sufficiency, suppose there exist $X \succ 0$, $A_i$, $B_i$, $C_i$, $D_i$ and $\hat{\mu },\hat{\zeta },\hat{\eta }>0$ such that (17) holds. By taking $\mu = \hat{\mu }$, $\zeta = \hat{\zeta }$, and $\eta = \hat{\eta }^{-1}$, we immediately conclude from [17, Proposition 3.9] that (16) holds for all $w_e\in \mathcal {L}_2$. Given the partitioning $w_e= \operatorname {col}(w_c,w_a,d_c,u_a)$ and $z_{cl} = \operatorname {col}(z_c-z_a,d_a)$, (16) can be equivalently written as (14). This, by Definition 2, indicates that $\boldsymbol{\Sigma }_a$ is a ($\gamma ,\delta$)-abstraction of $\boldsymbol{\Sigma }_c$. $\square$

#### Remark 4

The inclusion of parameter $\varepsilon$ in (14) enables us to derive the representation (16) which is then characterized as (17). In fact, the presence of $\varepsilon$ in (16) is crucial for the necessity of (17), see [17, Proposition 3.9] for details. $\triangle$

Proposition 2 characterizes ($\gamma ,\delta$)-abstraction as the feasibility of the *nonlinear* matrix inequality (17), where $A_i$, $B_i$, $C_i$, $D_i$, and *X* appear as unknown variables. However, by eliminating $A_i$, $B_i$, $C_i$, and $D_i$ using an elimination of variables technique (see, e.g., [22, 25]), we equivalently express (17) in terms of an LMI. The following result characterizes ($\gamma ,\delta$)-abstraction as an LMI feasibility problem that is solely in terms of the parameters of $\boldsymbol{\Sigma }_c$ and $\boldsymbol{\Sigma }_a$.

#### Theorem 1

For $\gamma ,\delta >0$, $\boldsymbol{\Sigma }_a$ is a ($\gamma ,\delta$)-abstraction of $\boldsymbol{\Sigma }_c$ if and only if there exist matrices $\Phi ,\Psi \succ 0$ and constants $\hat{\mu },\hat{\zeta },\hat{\eta }>0$ such that

18a $$\begin{aligned} \begin{bmatrix} \mathcal {N}_\Phi & 0\\ 0& I \end{bmatrix}^\top \begin{bmatrix} \begin{array}{c|c} \begin{matrix} \langle \Phi A_e\rangle _s& \Phi E_e\\ E_e^\top \Phi & -Q(\hat{\mu },\hat{\zeta }) \end{matrix}& \begin{matrix} H_e^\top \\ 0 \end{matrix}\\ \hline \begin{matrix} H_e &  \qquad \quad 0 \end{matrix}&-R(\hat{\eta }) \end{array} \end{bmatrix}\begin{bmatrix} \mathcal {N}_\Phi & 0\\ 0& I \end{bmatrix}\prec 0, \end{aligned}$$

18b $$\begin{aligned} \begin{bmatrix} \mathcal {N}_\Psi & 0\\ 0& I \end{bmatrix}^\top \begin{bmatrix} \begin{array}{c|c} \begin{matrix} \langle A_e\Psi \rangle _s &  \Psi H_e^\top \\ H_e \Psi & -R(\hat{\eta }) \end{matrix}& \begin{matrix} E_e\\ 0 \end{matrix}\\ \hline \begin{matrix} \quad E_e^\top \quad & \qquad \quad 0 \end{matrix}&\begin{matrix} -Q(\hat{\mu },\hat{\zeta }) \end{matrix} \end{array} \end{bmatrix}\begin{bmatrix} \mathcal {N}_\Psi & 0\\ 0& I \end{bmatrix}\prec 0, \end{aligned}$$

18c $$\begin{aligned} \begin{bmatrix} \Phi & I\\ I& \Psi \end{bmatrix}\succcurlyeq 0, \end{aligned}$$

where $A_e$, $B_e$, $E_e$, $C_e$, $D_e$, $H_e$, and $K_e$ are given by (11), *Q* and *R* are given as in (15), $$ \mathcal {N}_\Phi = \begin{bmatrix} C_e&D_e \end{bmatrix}^\perp $$, and $$\mathcal {N}_\Psi = \begin{bmatrix} B_e^\top&K_e^\top \end{bmatrix}^\perp $$. In this case, (14) is satisfied with $\mu = \hat{\mu }$, $\zeta = \hat{\zeta }$, and $\eta = \hat{\eta }^{-1}$.

#### Proof

*If part:* We show that the feasibility of (18) implies the existence of matrices $X\succ 0$, $A_i$, $B_i$, $C_i$, $D_i$, and constants $\mu ,\zeta ,\eta >0$ such that (17) holds. We then utilize Proposition 2 to conclude that $\boldsymbol{\Sigma }_a$ is a ($\gamma ,\delta$)-abstraction of $\boldsymbol{\Sigma }_c$.

We follow a similar procedure to that of the $H_\infty$ synthesis problem (see, e.g., [25, Theorem 4.3]) to conclude the existence of $A_i$, $B_i$, $C_i$, and $D_i$. Suppose there exist $\Phi ,\Psi \succ 0$ and $\hat{\mu },\hat{\zeta },\hat{\eta }>0$ such that (18) holds. Considering (18c), it follows from [22, Lemma 7.8] that there exist matrices $X_2,Y_2,X_3,Y_3\in \mathbb {R}^{(n_c+n_a)\times (n_c+n_a)}$ such that

19 $$\begin{aligned} \begin{bmatrix} \Phi & X_2\\ X_2^\top & X_3 \end{bmatrix} \succ 0,\quad \begin{bmatrix} \Psi & Y_2\\ Y_2^\top & Y_3 \end{bmatrix} = \begin{bmatrix} \Phi & X_2\\ X_2^\top & X_3 \end{bmatrix}^{-1}. \end{aligned}$$

We use $X_2$ and $X_3$ to define the matrices

20 $$\begin{aligned} \begin{aligned} \Lambda&= \begin{bmatrix} \langle \Phi A_e\rangle _s&  A_e^\top X_2& \Phi E_e& H_e^ \top \\ X_2^\top A_e& 0& X_2^\top E_e& 0\\ E_e^\top \Phi & E_e^\top X_2& -Q(\hat{\mu },\hat{\zeta })& 0\\ H_e& 0& 0& -R(\hat{\eta }) \end{bmatrix},\\ U&= \begin{bmatrix} X_2^\top & X_3& 0& 0\\ B_e^\top \Phi & B_e^\top X_2^\top & 0& K_e^\top \end{bmatrix},\\ V&=\begin{bmatrix} 0& I& 0& 0\\ C_e& 0& D_e& 0 \end{bmatrix}. \end{aligned} \end{aligned}$$

Defining

21 $$\begin{aligned} X = \begin{bmatrix} \Phi & X_2\\ X_2^\top & X_3 \end{bmatrix}, \quad Y = \begin{bmatrix} \Psi & Y_2\\ Y_2^\top & Y_3 \end{bmatrix}, \end{aligned}$$

we follow similar steps as in [25, Theorem 4.2] to obtain

$$\begin{aligned} \begin{aligned} V^\perp&= \begin{bmatrix} I& 0& 0& 0\\ 0& 0& I& 0\\ 0& I& 0& 0\\ 0& 0& 0& I \end{bmatrix} \begin{bmatrix} 0& 0& I& 0\\ C_e& D_e& 0& 0 \end{bmatrix}^\perp = \begin{bmatrix} \begin{array}{c|c} \begin{matrix} I& 0\\ 0& 0\\ 0& I\\ 0& 0 \end{matrix}& \begin{matrix} 0& 0\\ I& 0\\ 0& 0\\ 0& I \end{matrix} \end{array} \end{bmatrix}\begin{bmatrix} \begin{array}{cc} \mathcal {N}_\Phi & \hspace{-3pt}0\\ \hline \begin{matrix} 0\\ 0 \end{matrix}& \begin{matrix} 0\\ I \end{matrix} \end{array} \end{bmatrix}, \end{aligned} \end{aligned}$$

and

$$\begin{aligned} \begin{aligned} U^\perp&= \begin{bmatrix} Y& 0\\ 0& I \end{bmatrix} \begin{bmatrix} I& 0& 0& 0\\ 0& 0& 0& I\\ 0& 0& I& 0\\ 0& I& 0& 0 \end{bmatrix} \begin{bmatrix} 0& 0& 0& I\\ B_e^\top & K_e^\top & 0& 0 \end{bmatrix}^\perp = \begin{bmatrix} Y& 0\\ 0& I \end{bmatrix}\begin{bmatrix} \begin{array}{c|c} \begin{matrix} I& 0\\ 0& 0 \end{matrix}& \begin{matrix} 0& 0\\ 0& I \end{matrix}\\ \hline \begin{matrix} 0& 0\\ 0& I \end{matrix}& \begin{matrix} I& 0\\ 0& 0 \end{matrix} \end{array} \end{bmatrix}\begin{bmatrix} \begin{array}{cc} \mathcal {N}_\Psi & \hspace{-2pt}0\\ \hline \begin{matrix} 0\\ 0 \end{matrix}& \begin{matrix} I\\ 0 \end{matrix} \end{array} \end{bmatrix}. \end{aligned} \end{aligned}$$

After performing a few arrangements, we may rewrite (18a) and (18b) as $V^{\perp \top }\Lambda V\prec 0$ and $U^{\perp \top }\Lambda U\prec 0$, respectively. It then follows from the so-called *projection* lemma (see, e.g., [25, Lemma 3.1]) that there exists a matrix $\Omega$ such that

22 $$\begin{aligned} \Lambda + U^\top \Omega V + V^\top \Omega ^\top U \prec 0. \end{aligned}$$

Partitioning $$\Omega $$ as

23 $$\begin{aligned} \Omega = \begin{bmatrix} A_i& B_i\\ C_i& D_i \end{bmatrix}, \end{aligned}$$

we immediately notice that (22) is equivalent to (17), *i.e.,* the feasibility problem (17) admits a solution. This, however, as a result of Proposition 2, implies that $\boldsymbol{\Sigma }_a$ is a ($\gamma ,\delta$)-abstraction of $\boldsymbol{\Sigma }_c$. Additionally, in this case, (14) is satisfied with $\mu = \hat{\mu }$, $\zeta = \hat{\zeta }$, and $\eta = \hat{\eta }^{-1}$.

*Only if part:* Suppose $\boldsymbol{\Sigma }_a$ is a ($\gamma ,\delta$)-abstraction of $\boldsymbol{\Sigma }_c$. It then follows from Proposition 2 that there exist $X\succ 0$, $A_i$, $B_i$, $C_i$, $D_i$, and $\hat{\mu },\hat{\zeta },\hat{\eta }>0$ such that (17) holds. We collect $A_i$, $B_i$, $C_i$, and $D_i$ into (23). We then define $Y=X^{-1}$ and partition *X*, *Y* according to $A_e$ and $A_i$ as

$$\begin{aligned} \begin{aligned} X&= \begin{bmatrix} X_1& X_2\\ X_2^\top & X_3 \end{bmatrix},&Y&= \begin{bmatrix} Y_1& Y_2\\ Y_2^\top & Y_3 \end{bmatrix}. \end{aligned} \end{aligned}$$

We then take $\Phi = X_1$, $\Psi = Y_1$, and construct the matrices $\Lambda$, *U*, and *V* as in (20). Accordingly, we write (17) as (22). It then follows from the projection lemma that $V^{\perp \top }\Lambda V\prec 0$ and $U^{\perp \top }\Lambda U\prec 0$, which can be respectively written as (18a) and (18b) with $\Phi = X_1$ and $\Psi = Y_1$. In addition, since $Y = X^{-1}$, it follows from [22, Lemma 7.8] that (18c) holds. $\square$

Theorem 1 characterizes ($\gamma ,\delta$)-abstraction as the feasibility problem (18). We exploit the solution of (18) to construct the interface (9).

### Interface design

Having characterized ($\gamma ,\delta$)-abstraction in terms of the LMI feasibility problem (18), we now construct the interface (9) on the basis of the solution of (18). Given the concrete system (7), the abstract system (8), and the constants $\gamma ,\delta >0$, we construct the interface (9) according to the following procedure, inspired by [25].

*Step 1:* We construct the matrices $A_e$, $B_e$, $C_e$, $D_e$, $E_e$, $H_e$, and $K_e$ as in (11). We then solve the feasibility problem (18) to obtain the matrices $\Phi$ and $\Psi$.

*Step 2:* As explained in the proof of Theorem 1, we make use of $\Phi$ and $\Psi$ to construct $X_2$, $Y_2$, $X_3$, and $Y_3$ such that (19) holds. Utilizing the the positive definiteness of $\Psi$, we take the Schur complement of (18c), which, as a result of [26, Proposition 8.2.4], implies that $\Phi -\Psi ^{-1} \succcurlyeq 0$. We then obtain $X_2$ as the *principal square root* (see, e.g., [26, Section 8.5]) of $\Phi -\Psi ^{-1}$, *i.e.,* $X_2$ is a positive semi-definite matrix that satisfies $X_2 X_2^\top = \Phi -\Psi ^{-1}$. After taking $X_3 = I$, we observe that $\Phi - X_2X_3^{-1}X_2^\top = \Psi ^{-1} \succ 0$. We then take Schur complement to conclude that

$$\begin{aligned} \begin{bmatrix} \Phi & X_2\\ X_2^\top & X_3 \end{bmatrix}\succ 0. \end{aligned}$$

*Step 3:* Having obtained $X_2$ and $X_3$, we construct the matrices $\Lambda$, *U*, and *V* as in (20). We then solve the feasibility problem (22) which is now linear in terms of the variable $\Omega$.

*Step 4:* We partition $\Omega$ as in (23) and obtain the matrices $A_i$, $B_i$, $C_i$, and $D_i$. It is worth mentioning that in this case, the interface is of order $n_i = n_c+n_a$.

## Abstract controller synthesis

Having identified the abstract system according to the notion of ($\gamma ,\delta$)-abstraction and having constructed the interface, we now formalize the synthesis problem for the abstract system. Given a specification $\boldsymbol{S}$, for $\gamma _a,\delta _a>0$, we aim to design an abstract controller $\boldsymbol{\Gamma }_a$ such that (1) the controlled abstract system is 0-asymptotically stable and (2) the specification $\boldsymbol{S}$ is ($\gamma _a,\delta _a$)-similar to the controlled abstract system. Such controller, however, must be designed such that the concrete controller (obtained by refinement) achieves 0-asymptotic stability and ($\gamma _c,\delta _c$)-similarity for the controlled concrete system. We guarantee this by imposing constraints on the abstract controller. We therefore design a *constrained* abstract controller $\boldsymbol{\Gamma }_a$ that achieves 0-asymptotic stability and ($\gamma _a,\delta _a$)-similarity for the controlled abstract system.

**Fig. 4:**
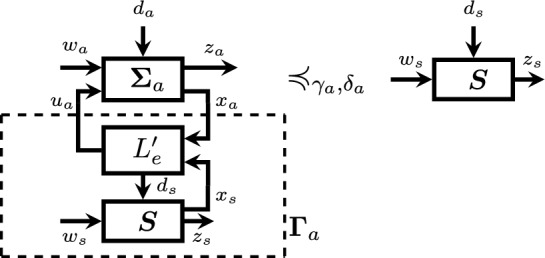
The abstract controller $\boldsymbol{\Gamma }_a$ can be regarded as the composite system that contains $\boldsymbol{S}$ and generates the abstract control input $u_a$ and the driving variable $d_s$

As the first step, we formally state the constrained synthesis problem for the abstract system. For this purpose, we specify the structure of the control input for the abstract system and the driving variable for the specification. Bearing in mind that the abstract system (8) and the specification (2) are *virtual* systems whose states are *always* available for measurement, we structure the abstract control input $u_a$ as

24 $$\begin{aligned} u_a = L_{a,1}x_a+L_{a,2}x_s, \end{aligned}$$

where $L_{a,1}\in \mathbb {R}^{m_a\times n_a}$ and $L_{a,2}\in \mathbb {R}^{m_a\times n_s}$, *i.e.,* we take $u_a$ as a static state feedback from the abstract system as well as the specification. Here, we recall that $x_s \in \mathbb {R}^{n_s}$ is the state of the specification system $\boldsymbol{S}$. In light of Remark 2, we may also take the driving variable for the specification system as a static state feedback

25 $$\begin{aligned} d_s = L_{s,1}x_a+L_{s,2}x_s, \end{aligned}$$

with $L_{s,1}\in \mathbb {R}^{q_s\times n_a}$ and $L_{s,2}\in \mathbb {R}^{q_s\times n_s}$. We now define $x_e':= \operatorname {col}(x_a,x_s)$, $u_e':= \operatorname {col}(u_a,d_s)$, $w_e':= \operatorname {col}(w_a,w_s,d_a)$, and $z_e':=\operatorname {col}(z_a-z_s,u_a,d_s)$ to collect the dynamics of $\boldsymbol{\Sigma }_a$ and $\boldsymbol{S}$ into the *extended* system

26 $$\begin{aligned} \begin{aligned} \dot{x}_e'&= A_e'x_e' + B_e'u_e'+E_e'w_e',\\ z_e'&=H_e'x_e'+K_e'u_e', \end{aligned} \end{aligned}$$

where

27 $$\begin{aligned} \begin{aligned} A_e'&= \begin{bmatrix} A_a& 0\\ 0& A_s \end{bmatrix},&B_e'&=\begin{bmatrix} B_a& 0\\ 0& F_s \end{bmatrix},&E_e'&=\begin{bmatrix} E_a& 0& F_a\\ 0& E_s& 0 \end{bmatrix},\\ H_e'&=\begin{bmatrix} H_a& -H_s\\ 0& 0\\ 0& 0 \end{bmatrix},&K_e'&=\begin{bmatrix} 0& 0\\ I& 0\\ 0& I \end{bmatrix}. \end{aligned} \end{aligned}$$

Then, after choosing $u_a$ and $d_s$ respectively according to (24) and (25), we obtain

28 $$\begin{aligned} \begin{aligned} \dot{x}_e'&= (A_e'+B_e'L_e')x_e'+E_e'w_e',\\ z_e'&=(H_e'+K_e'L_e')x_e', \end{aligned} \end{aligned}$$

where

29 $$\begin{aligned} L_e' = \begin{bmatrix} L_{a,1}& L_{a,2}\\ L_{s,1}& L_{s,2} \end{bmatrix}. \end{aligned}$$

On the basis of (28), we formally state the constrained abstract synthesis problem as follows.

*Constrained Abstract Synthesis Problem:* Given a specification $\boldsymbol{S}$, an abstract system $\boldsymbol{\Sigma }_a$, and constants $\gamma _a,\delta _a,\mu _a,\zeta _a>0$, find the matrix (29) such that (28) is 0-asymptotically stable and there exist constants $\varepsilon _a,\eta _a>0$ such that for every external input $w_a,w_s\in \mathcal {L}_2$ and every driving signal $d_a\in \mathcal {L}_2$,

30 $$\begin{aligned} \left\Vert z_a-z_s\right\Vert ^2 \le&\ \gamma _a\left\Vert w_a-w_s\right\Vert ^2+(\delta _a-\varepsilon _a)\left\Vert \begin{bmatrix} w_a\\ w_s \end{bmatrix}\right\Vert ^2 \\&+(\mu _a-\varepsilon _a)\left\Vert d_a\right\Vert ^2-\zeta _a\left\Vert u_a\right\Vert ^2 - \eta _a\left\Vert d_s\right\Vert ^2.\nonumber \end{aligned}$$

We claim that a matrix $L_e'$ solving the constrained abstract synthesis problem leads to an abstract controller such that the specification $\boldsymbol{S}$ is ($\gamma _a,\delta _a$)-similar to the controlled abstract system. Namely, the system (28) can be viewed as the interconnection of $\boldsymbol{\Sigma }_a$ and $\boldsymbol{S}$ through $L_e'$ (using (24) and (25)), as illustrated in Fig. 4. Then, we regard the composition of $L_e'$ and $\boldsymbol{S}$ as the abstract controller $\boldsymbol{\Gamma }_a$, *i.e.,* Fig. 4 gives the controlled abstract system that comprises $\boldsymbol{\Sigma }_a$ and $\boldsymbol{\Gamma }_a$. Then, it follows from (30) that for every $w_a,w_s\in \mathcal {L}_2$ and every $d_a\in \mathcal {L}_2$, there exists a $d_s$ (namely, the one generated through (25)) such that

$$\begin{aligned} \left\Vert z_a-z_s\right\Vert ^2 \le \gamma _a\left\Vert w_a-w_s\right\Vert ^2+(\delta _a-\varepsilon _a)\left\Vert \begin{bmatrix} w_a\\ w_s \end{bmatrix}\right\Vert ^2+(\mu _a-\varepsilon _a)\left\Vert d_a\right\Vert ^2- \eta _a\left\Vert d_s\right\Vert ^2. \end{aligned}$$

In other words, $\boldsymbol{S}$ is ($\gamma _a,\delta _a$)-similar to the controlled abstract system, recall Definition 1. It is worth noting that the abstract controller $\boldsymbol{\Gamma }_a$ is constrained in the sense that it is required to generate a $u_a$ that satisfies (30). In the following result, by characterizing the existence of $L_e'$, we give a characterization of an abstract controller that solves the constrained abstract synthesis problem.

### Theorem 2

For $\gamma _a,\delta _a,\mu _a,\zeta _a>0$, there exists a matrix $L_e'$ such that (28) is 0-asymptotically stable and there exist constants $\varepsilon _a,\eta _a>0$ such that for every external input $w_a,w_s\in \mathcal {L}_2$ and every driving signal $d_a\in \mathcal {L}_2$, (30) holds if and only if there exist matrices $Y\succ 0$, $\Pi$, and a scalar $\hat{\eta }_a>0$ such that

31 $$\begin{aligned} \begin{bmatrix} \langle A_e'Y+B_e'\Pi \rangle _s& E_e'& (H_e'Y+K_e'\Pi )^\top \\ E_e^{'\top }& -Q(\mu _a)& 0\\ H_e'Y+K_e'\Pi & 0& -R(\zeta _a^{-1},\hat{\eta }_a) \end{bmatrix}\prec 0, \end{aligned}$$

where

32 $$\begin{aligned} Q(\mu _a)&= \begin{bmatrix} (\gamma _a+\delta _a)I& -\gamma _a I& 0\\ -\gamma _a I&  (\gamma _a+\delta _a)I& 0\\ 0& 0& \mu _a I \end{bmatrix},&R(\zeta _a^{-1},\hat{\eta }_a)&= \begin{bmatrix} I& 0& 0\\ 0& \zeta _a^{-1} I& 0\\ 0& 0& \hat{\eta }_a I \end{bmatrix}. \end{aligned}$$

In this case, (30) is satisfied with $\eta _a = \hat{\eta }_a^{-1}$.

### Proof

*If part:* Suppose there exist $Y\succ 0$, $\Pi$, and $\varepsilon _a,\hat{\eta }_a>0$ such that (31) holds. Then, after defining $L_e'= \Pi Y^{-1}$, we easily observe that (31) is equivalent to

33 $$\begin{aligned} \begin{bmatrix} \langle Y^{-1}(A_e'+B_e'L_e')\rangle _s& Y^{-1}E_e'& (H_e'+K_e'L_e')^\top \\ E_e^{'\top } Y^{-1}& -Q(\mu _a)& 0\\ H_e'+K_e'L_e'& 0& -R(\zeta _a^{-1},\hat{\eta }_a) \end{bmatrix}\prec 0. \end{aligned}$$

This, as a result of [17, Proposition 3.9], implies that $L_e'$ is such that (28) is 0-asymptotically stable and there exists an $\varepsilon _a>0$ such that for every $w_a,w_s,d_s\in \mathcal {L}_2$,

34 $$\begin{aligned} \begin{aligned} {{\int }}_{0}^\infty \begin{bmatrix} w_e'(\tau )\\ z_e'(\tau ) \end{bmatrix}^\top \begin{bmatrix} -Q(\mu _a)+\varepsilon I& 0\\ 0& R(\zeta _a,\hat{\eta }_a^{-1}) \end{bmatrix}\begin{bmatrix} w_e'(\tau )\\ z_e'(\tau ) \end{bmatrix}\textrm{d}\tau \le 0 \end{aligned} \end{aligned}$$

which, after taking $\eta _a = \hat{\eta }_a^{-1}$, can be equivalently written as (30).

*Only if part:* Suppose there exists $L_e'$ such that (28) is 0-asymptotically stable and there exist $\eta _a,\varepsilon _a>0$ such that for every $w_a,w_s,d_s\in \mathcal {L}_2$, (30) holds. After defining $Q(\mu _a)$ and $R(\zeta _a,\eta _a^{-1})$ as in (32), we rewrite (30) as (34). Taking $\hat{\eta }_a = \eta _a^{-1}$, we conclude from [17, Proposition 3.9] that there exists $Y\succ 0$ such that (33) holds, which is equivalent to (31). $\square$

According to Theorem 2, the feasibility of (31) guarantees the existence of the constrained abstract controller $\boldsymbol{\Gamma }_a$. The solution of (31) can be utilized to construct $\boldsymbol{\Gamma }_a$. In fact, by defining $L_e'= \Pi Y^{-1}$, we may construct $\boldsymbol{\Gamma }_a$ as the composite system that contains $\boldsymbol{S}$ and generates $u_a$ and $d_s$ respectively according to (24) and (25), see Fig. 4.

## Concrete controller synthesis

In this section, under the assumption that the interface $\boldsymbol{I}$ and the abstract controller $\boldsymbol{\Gamma }_a$ are obtained (respectively as in Sects. 4 and 5), we now characterize a condition which guarantees that the concrete controller (obtained as in Fig. 2) achieves conditions (I) and (II). For this purpose, we first conduct a *stability analysis* to characterize the constraints on the abstract controller such that, after refinement, the resulting concrete controller $\boldsymbol{\Gamma }_c$ achieves condition (I), *i.e.,* the closed-loop system that contains $\boldsymbol{\Sigma }_c$ and $\boldsymbol{\Gamma }_c$ is 0-asymptotically stable. Then, we *parameterize* $\gamma _a,\delta _a>0$ in such a way that this concrete controller also achieves condition (II). We accordingly propose an algorithm for hierarchical control of $\boldsymbol{\Sigma }_c$.

As the first step, we obtain the controlled concrete system that contains $\boldsymbol{\Sigma }_c$, $\boldsymbol{\Sigma }_i$, $\boldsymbol{\Sigma }_a$, $L_e'$, and $\boldsymbol{S}$ (as illustrated in Fig. 2) in terms of the parameters (12) and (28). After defining the matrices $$P_1=\begin{bmatrix} 0&0&0&I \end{bmatrix}^\top $$, $$P_2=\begin{bmatrix} I&0 \end{bmatrix}$$, and $$P_3=\begin{bmatrix} 0&I \end{bmatrix}$$, we obtain the dynamics of the controlled concrete system as

35 $$\begin{aligned} \begin{aligned} \begin{bmatrix} \dot{x}_c\\ \dot{x}_a\\ \dot{x}_i\\ \dot{x}_s \end{bmatrix}&= \begin{bmatrix} A_{11}& A_{12}+A_{12}'& A_{13}& A_{14}\\ A_{21}& A_{22}+A_{22}'& A_{23}& A_{24}\\ A_{31}& A_{32}+A_{32}'& A_{33}& A_{34}\\ 0& A_{42}& 0& A_{44}\\ \end{bmatrix}\begin{bmatrix} x_c\\ x_a\\ x_i\\ x_s \end{bmatrix} + \begin{bmatrix} E_c& 0& F_c& 0\\ 0& E_a& 0& 0\\ 0& 0& 0& 0\\ 0& 0& 0& E_s \end{bmatrix}\begin{bmatrix} w_c\\ w_a\\ d_c\\ w_s \end{bmatrix},\\ z_c&= \begin{bmatrix} H_c&0&0&0 \end{bmatrix}\begin{bmatrix} x_c\\ x_i\\ x_a\\ x_s \end{bmatrix}, \end{aligned} \end{aligned}$$

where

$$\begin{aligned} \begin{aligned}&\begin{bmatrix} A_{11}& A_{12}& A_{13}\\ A_{21}& A_{22}& A_{23}\\ A_{31}& A_{32}& A_{33}\\ \end{bmatrix}= A_{cl},&\begin{bmatrix} A_{12}'& A_{14}\\ A_{22}'& A_{24}\\ A_{32}'& A_{34} \end{bmatrix}=E_{cl}P_1P_2L_e', \\&\begin{bmatrix} A_{42}&A_{44} \end{bmatrix}=P_3(A_e'+B_e'L_e'), \end{aligned} \end{aligned}$$

We therefore conduct stability analysis to derive a condition that guarantees 0-asymptotic stability of (35). Then, for given $\gamma _c,\delta _c>0$, we parameterize $\gamma _a,\delta _a>0$ in such a way that the specification $\boldsymbol{S}$ is ($\gamma _c,\delta _c$)-similar to (35).

### Stability analysis

**Fig. 5:**
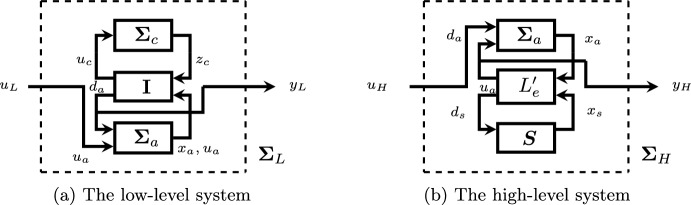
The low-level system $\boldsymbol{\Sigma }_L$ that contains $\boldsymbol{\Sigma }_c$, $\boldsymbol{I}$, and $\boldsymbol{\Sigma }_a$ is obtained from the closed-loop dynamics (12), whereas the high-level system $\boldsymbol{\Sigma }_H$ that contains $\boldsymbol{\Sigma }_a$, $L_e'$, and $\boldsymbol{S}$ is obtained from the extended dynamics (26)

We address the 0-asymptotic stability of (35) in terms of the asymptotic stability of the feedback interconnection of two 0-asymptotically stable systems with bounded $\mathcal {L}_2$ gains (see, e.g., [18]), namely a *low-level* system that is obtained from the closed-loop dynamics (12) and a *high-level* system that is obtained from the extended dynamics (26), see Fig. 5. In fact, this interconnected system may be regarded as an ‘expanded’ version of (35) in the sense that it comprises a low-level system that contains the concrete system $\boldsymbol{\Sigma }_c$, the interface $\boldsymbol{I}$, and the abstract system $\boldsymbol{\Sigma }_a$ and a high-level system that contains the abstract system $\boldsymbol{\Sigma }_a$, the constrained abstract controller $\boldsymbol{\Gamma }_a$, and the specification $\boldsymbol{S}$. We derive a sufficient condition for the asymptotic stability of this interconnected system, which then affords us with a condition that guarantees the 0-asymptotic stability of (35).

We utilize the closed-loop system (12) to construct the low-level system. Taking $w_c = 0$, $w_a = 0$, and $d_c = 0$, we define $x_L = x_{cl}$, $u_L = u_a$, and $y_L = d_a$ to obtain the dynamics

36 $$\begin{aligned} \boldsymbol{\Sigma }_L: \left\{ \begin{aligned} \dot{x}_L&= A_Lx_L + B_Lu_L,\\ y_L&= C_Lx_L+D_Lu_L, \end{aligned}\right. \end{aligned}$$

where $A_L = A_{cl}$, $B_L = E_{cl}M_L$, $C_L = N_LH_{cl}$, $D_L = N_LG_{cl}M_L$, $$M_L = \begin{bmatrix} 0&0&0&I \end{bmatrix}^\top $$, and $$N_L = \begin{bmatrix} 0&I \end{bmatrix}$$. The following result characterizes a bound on the $\mathcal {L}_2$ gain of the low-level system (36).

#### Proposition 3

For $\gamma ,\delta >0$, suppose the dynamics (12) is 0-asymptotically stable and there exist $\varepsilon ,\mu ,\zeta ,\eta >0$ such that for every $w_c,w_a,u_a,d_c\in \mathcal {L}_2$, (14) holds.Then, the dynamics (36) is 0-asymptotically stable and

37 $$\begin{aligned} \exists \varepsilon _L>0, \forall u_L\in \mathcal {L}_2: \; \left\Vert y_L\right\Vert ^2 \le \left( \frac{\zeta }{\eta }-\varepsilon _L\right) \left\Vert u_L\right\Vert ^2, \end{aligned}$$

where $y_L$ is the solution to (36) with $x_L(0) = 0$.

#### Proof

Since (12) is 0-asymptotically stable, the state matrix $A_{cl}$ is Hurwitz, which then implies the 0-asymptotic stability of (36). More importantly, by taking $w_c = 0$, $w_a = 0$, and $d_c = 0$, we conclude from (14) that (37) holds for $\varepsilon _L = \frac{\varepsilon }{\eta }$. $\square$

We now use the closed-loop system (28) to construct the high-level system. After taking $w_a = 0$ and $w_s = 0$, we define $x_H = x_e'$, $u_H = d_a$, and $y_H = u_a$ to obtain the dynamics

38 $$\begin{aligned} \boldsymbol{\Sigma }_H: \left\{ \begin{aligned} \dot{x}_H&= A_Hx_H+B_Hu_H,\\ y_H&= C_Hx_H, \end{aligned} \right. \end{aligned}$$

where $A_H = A_e'+B_e'L_e'$, $E_H = E_e'M_H$, $C_H = N_H(H_e'+K_e'L_e')$, $$M_H = \begin{bmatrix} 0&0&I \end{bmatrix}^\top $$, and $$N_H= \begin{bmatrix} 0&I&0 \end{bmatrix}$$. The following result then characterizes a bound on the $\mathcal {L}_2$ gain of the high-level system (38).

#### Proposition 4

For $\gamma _a,\delta _a,\mu _a,\zeta _a>0$, suppose that the dynamics (28) is 0-asymptotically stable and there exist $\varepsilon _a,\eta _a>0$ such that for every $w_a,w_s,d_a\in \mathcal {L}_2$, (30) holds. Then, the dynamics (38) is 0-asymptotically stable and

39 $$\begin{aligned} \exists \varepsilon _H>0, \forall u_H\in \mathcal {L}_2: \; \left\Vert y_H\right\Vert ^2 \le \left( \frac{\mu _a}{\zeta _a}-\varepsilon _H\right) \left\Vert u_H\right\Vert ^2, \end{aligned}$$

where $y_H$ is the solution to (38) with $x_H(0) = 0$.

#### Proof

It follows from 0-asymptotic stability of (28) that the matrix $A_e'+B_e'L_e'$ is Hurwitz, which, in turn, implies that (38) is 0-asymptotically stable. Moreover, by taking $w_a = 0$ and $w_s = 0$, we conclude from (30) that (39) holds for $\varepsilon _H = \frac{\varepsilon _a}{\zeta _a}$. $\square$

Having constructed the low-level and the high-level system, we construct the feedback interconnection of the low-level system $\boldsymbol{\Sigma }_L$ and the high-level system $\boldsymbol{\Sigma }_H$, see Fig. 5. By choosing $u_L = y_H$ and $u_H = y_L$, we obtain the feedback interconnection of $\boldsymbol{\Sigma }_L$ and $\boldsymbol{\Sigma }_H$ as

40 $$\begin{aligned} \begin{aligned} \begin{bmatrix} \dot{x}_L\\ \dot{x}_H \end{bmatrix} = \begin{bmatrix} A_L& B_LC_H\\ B_HC_L& A_H+B_HD_LC_H \end{bmatrix}\begin{bmatrix} x_L\\ x_H \end{bmatrix}. \end{aligned} \end{aligned}$$

In the following lemma, we obtain a small-gain condition that guarantees the asymptotic stability of (40).

#### Lemma 1

Suppose $\boldsymbol{\Sigma }_L$ and $\boldsymbol{\Sigma }_H$ are 0-asymptotically stable and respectively establish (37) and (39). Then, the dynamics (40) is asymptotically stable if

41 $$\begin{aligned} \frac{\zeta \mu _a}{\eta \zeta _a}\le 1. \end{aligned}$$

#### Proof

Given (37), it follows from [27, Definition 6.7] that $\boldsymbol{\Sigma }_L$ is finite-gain stable with gain $\frac{\zeta }{\eta }-\varepsilon _L$. Similarly, it follows from (39) that $\boldsymbol{\Sigma }_H$ is finite-gain-stable with gain $\frac{\mu _a}{\zeta _a}-\varepsilon _H$. Bearing in mind that $\varepsilon _L,\varepsilon _H>0$, it follows from (41) that

$$\begin{aligned} \left( \frac{\zeta }{\eta }-\varepsilon _L\right) \left( \frac{\mu _a}{\zeta _a}-\varepsilon _H\right) < 1, \end{aligned}$$

which, by [27, Corollary 9.5], implies that (40) is asymptotically stable. $\square$

We now utilize the feedback interconnection (40) to address the 0-asymptotic stability of (35). The following result exploits Lemma 1 to derive a condition that guarantees the 0-asymptotic stability of (35).

#### Theorem 3

For $\gamma ,\delta ,\gamma _a,\delta _a,\mu _a,\zeta _a>0$, suppose the assumptions in Propositions 3 and 4 hold. Then, the dynamics (35) is 0-asymptotically stable if (41) is satisfied.

#### Proof

It follows from Proposition 3 and Proposition 4 that $\boldsymbol{\Sigma }_L$ and $\boldsymbol{\Sigma }_H$ are 0-asymptotically stable and respectively establish (37) and (38). We now suppose that $\zeta$, $\eta$, $\zeta _a$, and $\mu _a$ are such that (41) holds. Then, it follows from Lemma 1 that (40) is asymptotically stable, *i.e.,* the matrix

42 $$\begin{aligned} \begin{bmatrix} A_L& B_LC_H\\ B_HC_L& A_H+B_HD_LC_H \end{bmatrix} \end{aligned}$$

is Hurwitz. We then write (42) in terms of the parameters of (35) as

$$\begin{aligned} A_L&= \begin{bmatrix} A_{11}& A_{12}& A_{13}\\ A_{21}& A_{22}& A_{23}\\ A_{31}& A_{32}& A_{33} \end{bmatrix},&B_LC_H&= \begin{bmatrix} A_{12}'& A_{14}\\ A_{22}'& A_{24}\\ A_{32}'& A_{34}\\ \end{bmatrix},\\ B_HC_L&=\begin{bmatrix} A_{21}& A_{22}-A_a& A_{23}\\ 0& 0& 0 \end{bmatrix},&A_H+B_HD_LC_H&= \begin{bmatrix} A_a+A_{22}'& A_{24}\\ A_{42}& A_{44} \end{bmatrix}. \end{aligned}$$

In order to prove the 0-asymptotic stability of (35), we show that the matrix

43 $$\begin{aligned} \begin{bmatrix} A_{11}& A_{12}+A_{12}'& A_{13}& A_{14}\\ A_{21}& A_{22}+A_{22}'& A_{23}& A_{24}\\ A_{31}& A_{32}+A_{32}'& A_{33}& A_{34}\\ 0& A_{42}& 0& A_{44}\\ \end{bmatrix} \end{aligned}$$

is Hurwitz. For this purpose, we show that every eigenvalue of (43) is an eigenvalue of (42). Let $\lambda$ be an eigenvalue of (43). Then, there exist vectors $v_1$, $v_2$, $v_3$, and $v_4$ such that

$$\begin{aligned} \begin{bmatrix} A_{11}& A_{12}+A_{12}'& A_{13}& A_{14}\\ A_{21}& A_{22}+A_{22}'& A_{23}& A_{24}\\ A_{31}& A_{32}+A_{32}'& A_{33}& A_{34}\\ 0& A_{42}& 0& A_{44}\\ \end{bmatrix}\begin{bmatrix} v_1\\ v_2\\ v_3\\ v_4 \end{bmatrix} = \lambda \begin{bmatrix} v_1\\ v_2\\ v_3\\ v_4 \end{bmatrix}. \end{aligned}$$

It is then straightforward to observe that

$$\begin{aligned} \begin{bmatrix} A_L& B_LC_H\\ B_HC_L& A_H+B_HD_LC_H \end{bmatrix}\begin{bmatrix} \begin{array}{c} v_1\\ v_2\\ v_3\\ \hline v_2\\ v_4 \end{array} \end{bmatrix} = \lambda \begin{bmatrix} v_1\\ v_2\\ v_3\\ v_2\\ v_4 \end{bmatrix}, \end{aligned}$$

*i.e.,* $\lambda$ is an eigenvalue of (42), which indicates that $\mathfrak {Re}\{\lambda \} < 0$. As a consequence, the matrix (43) is Hurwitz, which, in turn, implies the 0-asymptotic stability of (35). $\square$

By deriving a condition that guarantees the 0-asymptotic stability of the controlled concrete system, Theorem 3 basically specifies (41) as a constraint on the abstract controller, which leads to a concrete controller that achieves condition (I).

### Parametrization

Now that we have characterized the constraint on $\boldsymbol{\Gamma }_a$ that leads to a $\boldsymbol{\Gamma }_c$ that achieves condition (I), we parameterize $\gamma _a,\delta _a>0$ in a way that such $\boldsymbol{\Gamma }_c$ also achieves condition (II). Specifically, for a specification $\boldsymbol{S}$ and given constants $\gamma _c,\delta _c>0$, we choose the constants $\gamma _a,\delta _a>0$ and accordingly design the abstract controller $\boldsymbol{\Gamma }_a$ in such a way that the concrete controller $\boldsymbol{\Gamma }_c$ (which is obtained after refinement) achieves conditions (I) and (II), *i.e.,* we conduct hierarchical control synthesis in a way that the controlled concrete system is 0-asymptotically stable and the specification $\boldsymbol{S}$ is ($\gamma _c,\delta _c$)-similar to the controlled concrete system. To accomplish this, we first choose an abstract system $\boldsymbol{\Sigma }_a$ that satisfies the following assumption.

*Assumption 1:* $\boldsymbol{\Sigma }_a$ is a ($\gamma ,\delta$)-abstraction of $\boldsymbol{\Sigma }_c$ for $0<\gamma <\frac{1}{2}\gamma _c$ and $0<\delta <\frac{1}{3}\delta _c$.

The Assumption 1 is in fact a *design choice* on the abstraction, *i.e.,* it specifies $\gamma$ and $\delta$ according to which abstraction should be performed. For an abstract system $\boldsymbol{\Sigma }_a$ obtained according to Assumption 1, we then use Theorem 1 to construct an interface $\boldsymbol{I}$ such that the dynamics (12) is 0-asymptotically stable and there exist $\varepsilon ,\mu ,\zeta ,\eta >0$ such that for every $w_c,w_a,u_a,d_c\in \mathcal {L}_2$, (14) holds. Then, for these $\gamma$, $\delta$, $\zeta$, and $\eta$, we choose $\gamma _a,\delta _a>0$ and utilize Theorem 2 to design $\boldsymbol{\Gamma }_a$ such that the following assumption holds.

*Assumption 2:* The abstract controller $\boldsymbol{\Gamma }_a$ solves the constrained abstract synthesis problem with $\gamma _a = 2\gamma _c-\gamma$, $\delta _a = \frac{1}{3}\delta _c-\delta$, $\mu _a = \eta$, and $\zeta _a = \zeta$, where $\zeta$ and $\eta$ result from the ($\gamma ,\delta$)-abstraction in (14).

Assumption 2 gives a *design choice* on abstract controller synthesis, *i.e.,* it specifies the constraints according to which control synthesis should be performed. For an abstract system $\boldsymbol{\Sigma }_a$ obtained according to Assumption 1 and an abstract controller $\boldsymbol{\Gamma }_a$ designed according to Assumption 2, the following result guarantees that the concrete controller, obtained as in Fig. 2, achieves conditions (I) and (II).

#### Theorem 4

Under Assumption 1 and Assumption 2, the dynamics (35) is 0-asymptotically stable and the specification $\boldsymbol{S}$ is ($\gamma _c,\delta _c$)-similar to (35).

#### Proof

Suppose Assumption 1 and Assumption 2 hold. Then, since $\zeta$, $\eta$, $\zeta _a$, and $\mu _a$ satisfy (41), it follows from Theorem 3 that (35) is 0-asymptotically stable. We now take $w_c,w_s\in \mathcal {L}_2$, $d_c\in \mathcal {L}_2$ and choose $d_a$, $u_a$, and $d_s$ respectively according to (9), (24), and (25). Then, for $w_a = \frac{1}{2}(w_c+w_s)$, it follows from (14) that

$$\begin{aligned} \begin{aligned} \left\Vert z_c-z_a\right\Vert ^2 \le&\ \frac{\gamma }{4}\left\Vert w_c-w_s\right\Vert ^2 +(\delta -\varepsilon )\left\Vert \begin{bmatrix} w_c\\ \frac{1}{2}(w_c+w_s) \end{bmatrix}\right\Vert ^2\\&+(\mu -\varepsilon )\left\Vert d_c\right\Vert ^2 + (\zeta -\varepsilon )\left\Vert u_a\right\Vert ^2 - \eta \left\Vert d_a\right\Vert ^2, \end{aligned} \end{aligned}$$

which, by the triangle and Cauchy-Schwartz inequalities, leads to

44 $$\begin{aligned} \left\Vert z_c-z_a\right\Vert ^2 \le&\ \frac{\gamma }{4}\left\Vert w_c-w_s\right\Vert ^2+(\delta -\varepsilon )\left( \frac{3}{2} \left\Vert w_c\right\Vert ^2 + \frac{1}{2}\left\Vert w_s\right\Vert ^2 \right) +(\mu -\varepsilon )\left\Vert d_c\right\Vert ^2 \nonumber \\&+ (\zeta -\varepsilon )\left\Vert u_a\right\Vert ^2 - \eta \left\Vert d_a\right\Vert ^2\nonumber \\ \le&\ \frac{\gamma }{4}\left\Vert w_c-w_s\right\Vert ^2+\frac{3}{2}(\delta -\varepsilon )\left\Vert \begin{bmatrix} w_c\\ w_s \end{bmatrix}\right\Vert ^2+(\mu -\varepsilon )\left\Vert d_c\right\Vert ^2 \nonumber \\&+ (\zeta -\varepsilon )\left\Vert u_a\right\Vert ^2 - \eta \left\Vert d_a\right\Vert ^2. \end{aligned}$$

For $u_a$, $w_a$, $d_a$, and $d_s$ as chosen above, it follows from Assumption 2 that

45 $$\begin{aligned} \begin{aligned} \left\Vert z_a-z_s\right\Vert ^2 \le&\ \frac{2\gamma _c-\gamma }{4}\left\Vert w_c-w_s\right\Vert ^2+\frac{3}{2}(\frac{\delta _c}{3}-\delta -\varepsilon _a)\left\Vert \begin{bmatrix} w_c\\ w_s \end{bmatrix}\right\Vert ^2\\&+(\eta -\varepsilon _a)\left\Vert d_a\right\Vert ^2- \zeta \left\Vert u_a\right\Vert ^2 - \eta _a\left\Vert d_s\right\Vert ^2. \end{aligned} \end{aligned}$$

After adding (44) and (45), we use the triangle inequality to conclude that

$$\begin{aligned} \begin{aligned} \left\Vert z_a-z_s\right\Vert ^2 \le&\ \gamma \left\Vert w_c-w_s\right\Vert ^2 + (\delta _c-\varepsilon _c)\left\Vert \begin{bmatrix} w_c\\ w_s \end{bmatrix}\right\Vert ^2+(2\mu -\varepsilon _c)\left\Vert d_c\right\Vert ^2 - 2\eta _a\left\Vert d_s\right\Vert ^2, \end{aligned} \end{aligned}$$

for some $\varepsilon _c>0$. This, by Definition 1, indicates that $\boldsymbol{S}$ is ($\gamma _c,\delta _c$)-similar to (35). $\square$

Theorem 4 characterizes the constraints on the abstract controller that enable the construction of a concrete controller (as in Fig. 2) which achieves conditions (I) and (II). Given the constraints specified in Theorem 4, we may now hierarchically control (7) according to Algorithm 1.

**Algorithm 1:**
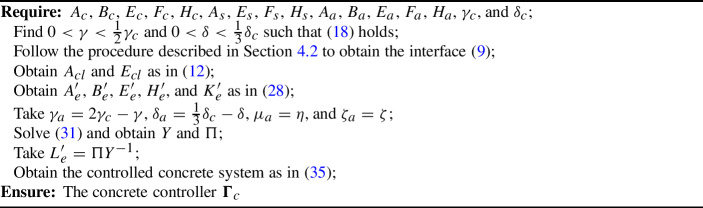
Hierarchical control of system (7)

#### Remark 5

Algorithm 1 requires the abstract system $\boldsymbol{\Sigma }_a$ to be given, *i.e.,* in order to utilize Algorithm 1, the designer must have constructed $\boldsymbol{\Sigma }_a$ in advance. We note that in many applications, the designer tends to approximately model an intricate concrete system with a simple one, whose behavior is well-studied, e.g., in electrical circuit design, engineers usually simplify intricate component models with simple components, such as resistor-inductor-capacitor (RLC) circuits. The designer may therefore regard such approximate model as an abstract system and accordingly utilize Theorem 1 to inspect whether it is a ($\gamma ,\delta$)-abstraction of the concrete system. Specifically, the designer may take $\gamma ,\delta >0$ as decision variables and minimize them with respect to the LMI constraint (18) such that the approximate model is a ($\gamma ,\delta$)-abstraction of the concrete system.

While designers may rely on their intuition and practical experience to construct the abstract system $\boldsymbol{\Sigma }_a$, they may also exploit the feasibility problem (17) to construct it. Specifically, one may consider the closed-loop dynamics (12), where in addition to parameters of the interface (*i.e.,* matrices $A_i$, $B_i$, $C_i$, and $D_i$), parameters of the abstract system (*i.e.,* $A_a$, $B_a$, $E_a$, $F_a$, and $H_a$) are taken as unknown decision variables to be determined. As a consequence, by following the procedure discussed in the proof of Proposition 2, one concludes that for $\gamma ,\delta >0$, there exists a ($\gamma ,\delta$)-abstraction of a given concrete system $\boldsymbol{\Sigma }_c$ if and only if there exist matrices $X\succ 0$, $A_i$, $B_i$, $C_i$, $D_i$, $A_a$, $B_a$, $E_a$, $F_a$, $H_a$ and constants $\hat{\mu },\hat{\zeta },\hat{\eta }>0$ such that (17) holds. One then utilizes elimination of variables and proceeds according to the proof of Theorem 1 to eliminate the unknown matrices, *i.e.,* the parameters of the abstract system and the interface. This leads to the characterization of the existence of a ($\gamma ,\delta$)-abstraction as the feasibility of a rank-constrained bilinear matrix inequality (BMI), whose solution then gives the abstract system. We note, however, that solving such a feasibility problem requires further investigation, which will be pursued in future work.

It follows from Algorithm 1 that hierarchical control of the concrete system (7) requires the solution of (18), which may be computationally expensive as its size increases proportionally to $n_c+n_s$. However, the benefit of hierarchical control according to Algorithm 1 is that it requires such expensive computations only once. In fact, such hierarchical scheme finds application in situations in which *multiple* control problems need to be solved for a *single* concrete system, *i.e.,* situations that necessitate the design of different controllers that establish distinct control objectives for a single concrete system. One such situation arises when a motor manufacturer provides an electrical motor for different users that utilize it for control problems with distinct specifications. In such situations, direct controller synthesis [23] is computationally prohibitive as it necessitates the *repetition* of computations that scale with the system/specification dimension. By contrast, hierarchical control synthesis is computationally affordable as it requires these expensive computations only *once*. We demonstrate this by comparing the computational costs of the direct and hierarchical synthesis methods in the following example.

#### Example 1

Consider a concrete system $\boldsymbol{\Sigma }_c$ that is utilized by users $\textrm{U}_1$, $\textrm{U}_2$, $\ldots$, $\textrm{U}_\ell$ for different purposes. Specifically, user $\textrm{U}_i$ aims to design a controller $\boldsymbol{\Gamma }_{c,i}$ such that

1. the controlled concrete system (*i.e.,* the closed-loop dynamics that contains $\boldsymbol{\Sigma }_c$ and $\boldsymbol{\Gamma }_{c,i}$) is 0-asymptotically stable;
2. a given specification $\boldsymbol{S}_i$ is ($\gamma _i,\delta _i$)-similar to the controlled concrete system for some $\gamma _i,\delta _i>0$.

Under the assumption that such controller exists, we utilize the direct and hierarchical control schemes to construct the controller and accordingly compare their computational costs.

We first suppose that each user $\textrm{U}_i$ directly synthesizes $\boldsymbol{\Gamma }_{c,i}$ (according to the technique proposed in [23]). This requires the solution of an LMI that scales proportionally to $n_c+n_{s_i}$, where $n_{s_i}$ denotes the dimension of $\boldsymbol{S}_i$. As a consequence, the overall computational cost for designing controllers $\boldsymbol{\Gamma }_{c,1}$, $\boldsymbol{\Gamma }_{c,2}$, $\ldots$, $\boldsymbol{\Gamma }_{c,\ell }$ increases proportionally to $\ell n_c + \sum _{i=1}^{\ell }n_{s_i}$.

We now let $\gamma ,\delta >0$ be such that $\gamma <\frac{1}{2}\gamma _i$ and $\delta <\frac{1}{3}\delta _i$ for all $i=1,2,\ldots ,\ell$. We accordingly let the low-dimensional abstract system $\boldsymbol{\Sigma }_a$ be a ($\gamma ,\delta$)-abstraction of $\boldsymbol{\Sigma }_c$. Inspecting this requires the solution of LMI (18), whose size increases proportionally to $n_c+n_a$. Based on the solution of (18), the interface $\boldsymbol{I}$ can be constructed according to the design procedure described in Sect. 4.2. We note that the abstract system $\boldsymbol{\Sigma }_a$ and the interface $\boldsymbol{I}$ may be constructed by the manufacturer of the concrete system $\boldsymbol{\Sigma }_c$, *i.e.,* the manufacturer of $\boldsymbol{\Sigma }_c$ takes the effort of finding $\boldsymbol{\Sigma }_a$ and solving the LMI (18) to construct $\boldsymbol{I}$. The manufacturer then makes the abstract system $\boldsymbol{\Sigma }_a$ and the interface $\boldsymbol{I}$ available to the users. We then suppose that each user $\textrm{U}_i$ utilizes these $\boldsymbol{\Sigma }_a$ and $\boldsymbol{I}$ to hierarchically synthesize $\boldsymbol{\Gamma }_{c,i}$ according to the proposed hierarchical scheme. This requires the solution of LMI (31) whose size increases proportionally to $n_a+n_{s_i}$. Thus the over all computational cost, which includes the cost of constructing $\boldsymbol{\Sigma }_a$ and $\boldsymbol{I}$ (performed by the manufacturer) and designing $\boldsymbol{\Gamma }_{c,1}$, $\boldsymbol{\Gamma }_{c,2}$, $\ldots$, $\boldsymbol{\Gamma }_{c,\ell }$ (performed by the users), increases proportionally to $n_c+(\ell +1)n_a + \sum _{i=1}^{\ell }n_{s_i}$. Clearly, when $n_a<<n_c$, the hierarchical scheme requires significantly less computations than the direct one.

We therefore observe that for a high-dimensional concrete system, the computational cost of hierarchical control according to Algorithm 1 is significantly less than that of the direct synthesis technique proposed in [23].

## Illustrative example

**Fig. 6:**
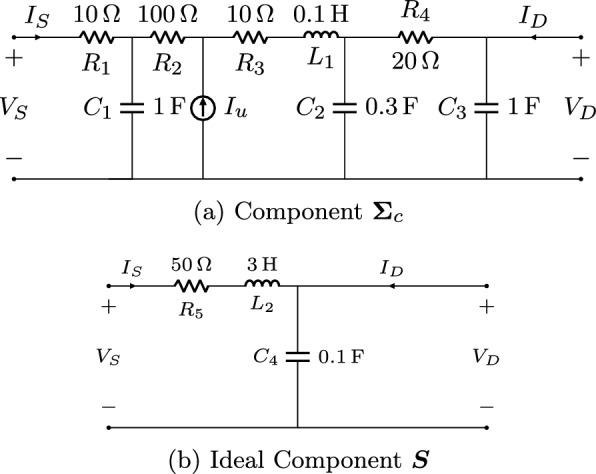
By applying the suitable current $I_u$, we control $\boldsymbol{\Sigma }_c$ to generate the voltage $v_{C_3}$ such that it approximates the ideal voltage $v_{C_4}$ generated by $\boldsymbol{S}$

We consider the hierarchical control of the electrical circuit depicted in Fig. 6a. Supplied by the source voltage $V_S$, the component $\boldsymbol{\Sigma }_c$ is controlled by the current source $I_u$ to feed a device with voltage $v_{C_3}$. The device, in turn, injects the unwanted current $I_D$ back into the component $\boldsymbol{\Sigma }_c$.

Suppose that the device *D* operates optimally when supplied by the voltage $v_{C_4}$ generated by the ‘ideal’ component $\boldsymbol{S}$, see Fig. 6b.

In order for the device to operate optimally, we control $v_{C_3}$ (by applying the suitable current $I_u$) such that it approximates the ideal voltage $v_{C_4}$. Regarding $\boldsymbol{\Sigma }_c$ as the concrete system and $\boldsymbol{S}$ as the specification, we first cast this problem into the framework of ($\gamma ,\delta$)-similarity. We then hierarchically design a controller that generates the suitable $I_u$.

Let $i_{L_i}$ and $v_{C_i}$ respectively denote the current passing through the inductor $L_i$ (for $i=1,2$) and the voltage across the capacitor $C_i$ (for $i=1,2,3,4$). Then, by taking $x_c = \operatorname {col}(v_{C_1}, i_{L_1}, v_{C_2}, v_{C_3})$, $u_c = I_u$, $w_c = V_S$, $d_c = I_D$, and $z_c = v_{C_3}$, we formulate $\boldsymbol{\Sigma }_c$ as in (7). Similarly, after taking $x_s = \operatorname {col}(i_{L_2}, v_{C_4})$, $w_s = V_S$, $d_s = I_D$, and $z_s = v_{C_4}$, we formulate $\boldsymbol{S}$ as in (2).

Due to environmental noises, the voltages with which $\boldsymbol{\Sigma }_c$ and $\boldsymbol{S}$ are supplied differ slightly, *i.e.,* the dissimilarity in the external inputs is negligible. However, such voltages may each have a large amplitude, *i.e.,* the external inputs may be large individually. For this reason, we choose $\gamma _c = 0.1$ and $\delta _c = 0.035$.

To hierarchically control $\boldsymbol{\Sigma }_c$, we consider the abstract system (8) with

46 $$\begin{aligned} A_a&= \begin{bmatrix} -3& 1\\ 0& -2 \end{bmatrix},&B_a&= \begin{bmatrix} 0.1\\ 0.4 \end{bmatrix},&E_a&= \begin{bmatrix} 0\\ 0.2 \end{bmatrix},&F_a&=\begin{bmatrix} 30\\ 0 \end{bmatrix},&C_a&= \begin{bmatrix} 1&0 \end{bmatrix}. \end{aligned}$$

It then follows from Theorem 1 that for $\gamma = 0.1$ and $\delta = 0.02$, the system (8), described by (46), is a ($\gamma ,\delta$)-abstraction of $\boldsymbol{\Sigma }_c$. Given that $\gamma <\frac{1}{2}\gamma _c$ and $\delta <\frac{1}{3}\delta _c$, we readily use Algorithm 1 to hierarchically control $\boldsymbol{\Sigma }_c$. The feasibility problems (18) and (31) are solved in MATLAB R2022b, using YALMIP [28] and SDPT3 [29].

**Fig. 7:**
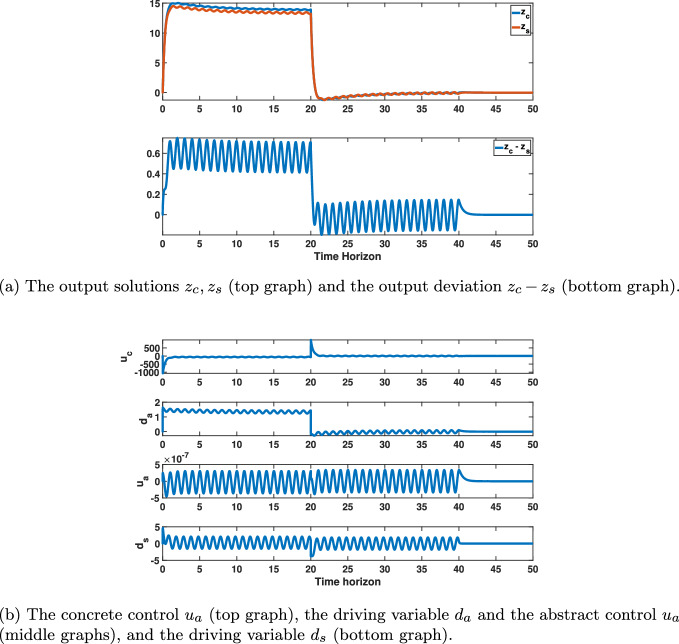
When the concrete control $u_c$ and the driving signal $d_a$ are provided by the interface (9), while the abstract control input $u_a$ and the driving variable $d_s$ are respectively chosen according to (24) and (25), the component $\boldsymbol{\Sigma }_c$ and the specification $\boldsymbol{S}$ admit similar output solutions

To evaluate the efficacy of the (hierarchically) synthesized concrete controller, we conduct a numerical simulation where the component $\boldsymbol{\Sigma }_c$ is fed with $w_c(t) = 100\sin (2\pi t)$ over the time interval [0, 40], while it is subject to $d_c(t) = 50$ over the [0, 20]. The abstract system $\boldsymbol{\Sigma }_a$ and the specification $\boldsymbol{S}$, on the other hand, are respectively fed with $w_a(t) = 100.1\sin (2\pi t)$ and $w_s(t) = 100.2\sin (2\pi t)$ over the time interval [0, 40]. We run the simulation over the period [0, 50], which is long enough for the systems to reach their steady state. The output solutions and the output deviations are depicted in Fig. 7a, whereas the control inputs and driving signals are illustrated in Fig. 7b.

It is clear from Fig. 7a that subject to a $d_s$ chosen according to (25), as depicted in the bottom graph of Fig. 7b, $\boldsymbol{S}$ reveals an output solution that is very similar to that of $\boldsymbol{\Sigma }_c$, which is subject to the $u_c$ generated by (9), as depicted in the top graph of Fig. 7b. In this case, the abstract system (8) is subject to $u_a$ and $d_a$ that are respectively chosen according to (24) and (9), as depicted in the middle graphs of Fig. 7b. Therefore, instead of direct controller synthesis for the high-dimensional system $\boldsymbol{\Sigma }_c$, we first synthesized a controller for the low-dimensional system (8) and then used the interface (9) to refine it into the concrete controller.

## Conclusion

In this paper, to compensate for the scalability issues in the synthesis of a dynamic controller that enforces ($\gamma ,\delta$)-similarity on the basis of a high-dimensional system model, we developed a hierarchical scheme to conduct controller synthesis. In this scheme, we identified abstractions according to the notion of ($\gamma ,\delta$)-abstraction, which measures to what extent the controlled concrete system behaves similarly to its abstraction. We utilized the $\mathcal {L}_2$ signal norm to measure behavioral similarity in terms of the sensitivity of the output difference to the input signals, and we characterized ($\gamma ,\delta$)-abstraction as an LMI feasibility problem whose solution then gives the interface. Subsequently, we studied the problem of abstract controller synthesis where we obtained the abstract controller as a solution of an LMI feasibility problem. After refining the abstract controller into the concrete one, we conducted an analysis to study the stability properties of the controlled concrete system. We finally proposed a step-by-step algorithm to conduct hierarchical control synthesis.

For future work, we will focus on using the notion of ($\gamma ,\delta$)-abstraction to construct abstract systems. We will also generalize the notion of ($\gamma ,\delta$)-similarity to nonlinear systems and accordingly extend the proposed hierarchical control scheme to nonlinear control systems. Lastly, by extending this framework to interconnected systems, we aim to conduct decentralized control synthesis for network systems.

## Data Availability

No datasets were generated or analysed during the current study.
